# Top-Down Machine Learning-Based Architecture for Cyberattacks Identification and Classification in IoT Communication Networks

**DOI:** 10.3389/fdata.2021.782902

**Published:** 2022-01-13

**Authors:** Qasem Abu Al-Haija

**Affiliations:** Department of Cybersecurity, Princess Sumaya University for Technology (PSUT), Amman, Jordan

**Keywords:** IoT communications, machine learning, shallow neural network, deep neural network, convolutional neural network, cyberattacks detection, systems engineering

## Abstract

With the prompt revolution and emergence of smart, self-reliant, and low-power devices, Internet of Things (IoT) has inconceivably expanded and impacted almost every real-life application. Nowadays, for example, machines and devices are now fully reliant on computer control and, instead, they have their own programmable interfaces, such as cars, unmanned aerial vehicles (UAVs), and medical devices. With this increased use of IoT, attack capabilities have increased in response, which became imperative that new methods for securing these systems be developed to detect attacks launched against IoT devices and gateways. These attacks are usually aimed at accessing, changing, or destroying sensitive information; extorting money from users; or interrupting normal business processes. In this research, we present new efficient and generic top-down architecture for intrusion detection, and classification in IoT networks using non-traditional machine learning is proposed in this article. The proposed architecture can be customized and used for intrusion detection/classification incorporating any IoT cyber-attack datasets, such as CICIDS Dataset, MQTT dataset, and others. Specifically, the proposed system is composed of three subsystems: feature engineering (FE) subsystem, feature learning (FL) subsystem, and detection and classification (DC) subsystem. All subsystems have been thoroughly described and analyzed in this article. Accordingly, the proposed architecture employs deep learning models to enable the detection of slightly mutated attacks of IoT networking with high detection/classification accuracy for the IoT traffic obtained from either real-time system or a pre-collected dataset. Since this work employs the system engineering (SE) techniques, the machine learning technology, the cybersecurity of IoT systems field, and the collective corporation of the three fields have successfully yielded a systematic engineered system that can be implemented with high-performance trajectories.

## Introduction

Internet of Things (IoT) is a global term to entitle the network of heterogeneous or homogenous resource-constrained physical objects (i.e., things) interrelated using diverse communication structures and topologies for the accomplishment of a common goal/application. These things are usually embedded with sensors, actuators, processors, software, and others for the purpose of transferring and communicating data with other devices over the Internet without necessitating the interaction of human-to-human (H2H) or human-to-computer (H2C) (Chiti et al., 2016). IoT is a promising disruptive technology with incredible growth and impact. It has been reported by Cisco Incorporation that more than 50 billion IoT devices are expected to be connected by 2021 (Evans, 2011).

Recently, the enormous technological revolution in electronics, computing, and sensing has offered essential infrastructure to develop today's wireless sensor networks (WSN) for different applications. The common theme of all these WSN systems is usually associated with the Internet of Things (IoT), where, through the use of sensors, the entire physical infrastructure is closely coupled with cyber world with its information and communication technologies (Al-Haija et al., 2017). IoT comprises a collection of heterogeneous resource-constrained devices interconnected *via* different network architectures, such as wireless sensor networks (WSN), machine-to-machine (M2M) networks, and cyber physical systems (CPS). Therefore, IoT has become the main standard for low-power lossy networks (LLNs) (Alrawais et al., 2017). However, the massive growth of IoT networks and applications, as the number of smart devices being deployed eventually reaches trillions, has created several unsafe environments that are highly vulnerable to security attacks. IoT security continues to be a serious issue as the number of security breaches is increasing from time to time. It has been reported that thousands of attacks are continuously emerging because of the addition of various services and protocols from IoT and seems to increase linearly (Al-Haija and Tawalbeh, 2019).

### Problem Background Review and Definition

The basic IoT-layered architecture is comprised of three layers: (A) the physical layer, where the different physical component “things,” such as sensors and actuators, are placed and connected to interact with the surrounding environment to gather information and perform data analytics about the designated phenomena. (B) the network layer, where the different communication and networking components, such as routers, switches, and gateways, are placed and connected to disclose and link the heterogeneous or homogenous elements of IoT system to communicate the data collected and sensed at the physical layer. (C) The application layer where the different services and applications, such as medical and eco-systems, are installed, configured, and deployed to process, compute, and store the corresponding data.

Commonly, most cyber-attacks are developed to target the application and network layers of the IoT system; for instance, Hyper Text Transfer Protocol (HTTP) Denial-of-Service (DoS) attack that targets the application layer (Paar and Pelzl, 2010). The HTTP DoS flooding attack aims to overwhelm a targeted server by flooding it with HTTP requests from multiple sources thus exploiting all the server processing capabilities, resulting in the authorized client request being denied. Similar types of other flood attacks include Synchronization/Transport Control Protocol (SYN/TCP) flood attack and the User Datagram Protocol (UDP) flood attack (Mahmoud et al., 2015). Other IoT cyber-attacks that target the application or network layers are as follows (Paar and Pelzl, 2010):

- Probe (side channel) attacks: attacks meant to investigate the network for well-known weakness or ports. Ping is a common utility for sending such a probe. Examples include SATAN and IPSWEEP attacks (Tavallaee et al., 2009).
- Denial-of-Service (DoS) attacks: attacks meant to fold down a system, device, or network, getting it unavailable to its legitimate users by overwhelming the target victim multiple sources. Examples include FLOODING, SMURF, and TAPDOOR attacks (Mahmoud et al., 2015).
- Root to Local (R2L) attacks: attacks meant to obtain illegal access to a victim system in the whole network (launched by an attacker who resides externally to the legal user network). Examples include FTPWRITE and IMAP attacks (Kolias et al., 2017).
- User to Root (U2R) attacks: attacks meant to earn the privileges of the network root (administrator level) by unauthorized user when legitimately accessing a local system (Launched by an attacker who has legal access with a non-privileged account). Examples include BUFFER_OVERFLOW and ROOTKIT attacks (Kolias et al., 2017).

It should be noted that the majority of IoT attacks are developed as slight deviations (i.e., mutations) of earlier known cyberattacks (Ambedkar and Kishore Babu, 2015). These slight mutations of these IoT attacks have been demonstrated to be difficult to identify/classify using traditional machine learning (TML) techniques (Sakurada and Yairi, 2014; Pongle and Chavan, 2015; Guy and Caspi, 2016; Kang and Kang, 2016; Wang et al., 2016; Sapre et al., 2019; Taher et al., 2019).

### Cyberattacks Mutaions

**Figure 1:**
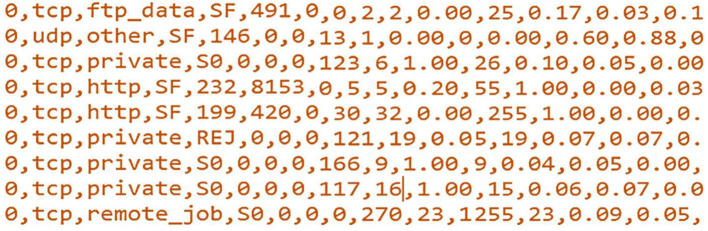
Sample records of the NSL-KDD training data set.

Like image recognition, in cybersecurity, more than 99% of new threats and malware are actually very small mutations of previously existing ones (Tavallaee et al., 2009). Indeed, cyberattacks/malware are also software, so they can also exhibit biological behavior as propagate, mutate, and replicate. This means that the newly developed cyberattacks are usually made by slightly changing the earlier known cyberattacks at very specific and minimal parts of the attack file/header, for instance, changing few bits of the attack/malware file. For example, Figure 1 shows sample records of the original NSL-KDD training dataset in the. csv format but read by Notepad in .txt format (prior to any processing technique). These samples are all classified as anomaly traffic records (the first feature corresponds to the class label, i.e., normal: 0 and anomaly: 1). One can mutate few bits of the depicted features to produce new anomaly traffic with new effect. This changing in the those few bits is termed as bit mutation. Therefore, traditional machine learning techniques have great difficulty in detecting a large portion of this new malware (newly mutated attacks).

### Research Goal and SOI Identification

**Figure 2:**
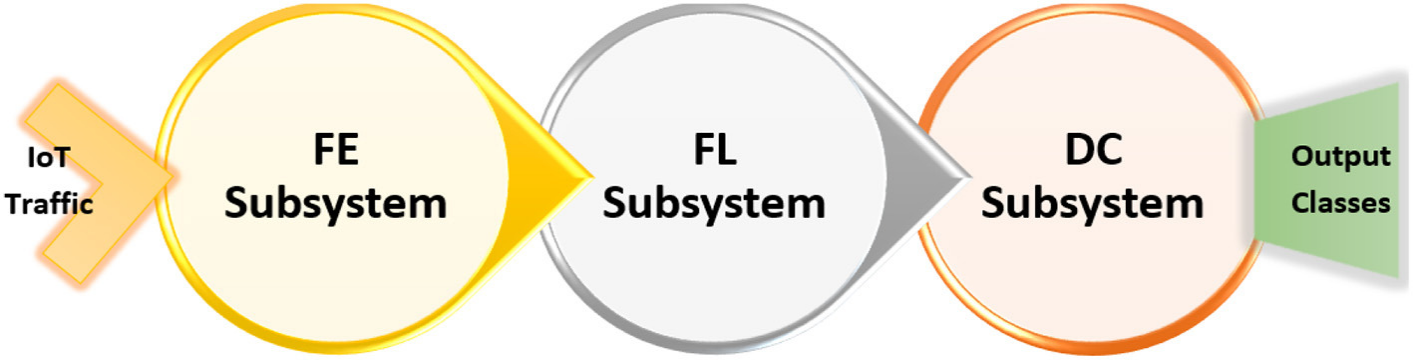
The three main subsystems composing the proposed System of Interest (SOI).

The goal of this research is the development of a system that can detect mutations of common IoT cyberattacks as discussed in the previous section using non-traditional machine learning methods. Thus, the system of interest (SOI: The acronym comes from INCOSE: International Council of System Engineering) in this research concerned with developing a software system at the IoT application layer to detect these mutated cyberattacks. Specifically, the SOI is composed of three subsystems (illustrated in Figure 2): Feature Engineering (FE) subsystem to handle preprocessing and encoding for the collected IoT traffic datasets to be fed as input features for the detection system, feature learning (FL) subsystem to train and test non-traditional machine learning (NML) algorithms for development of the learning module, and Detection and Classification (DC) subsystem to generate categorization for every IoT packet through either a two-class classifier (i.e., intrusion detection) or a multi-class classifier (intrusion classification). Besides, the developed system performance shall be evaluated *via* several metrics, such as accuracy, precision, sensitivity, specificity, false alarm rate, and others. Finally, the developed system shall be validated in comparison to IoT-Security-based machine learning (TML) applications.

Indeed, the novelty of this design focuses on the design methodology, which employs the top-down decomposing techniques for system development according to the systems engineering paradigm/life cycle, which realize the investigation of every subsystem/module, taking into consideration the different design alternatives corresponding to each individual module/subsystem. The systematic study of this paper has been developed by applying the systems engineering techniques to develop intelligent cybersecurity solutions for IoT systems. To the best of our knowledge, this is one of the few studies that provide a systematic design/architecture for the intrusion detection systems (IDSs) of IoT communication networks.

### System of Interest Technology Feasibility Analysis

This section discusses the existence of technologies and/or methods for development of the SOI. Methodologies for the development of the FE subsystem include Integer Encoding (INE), One Hot Encoding (OHE), Categorical Embedding Encoding (CEE), Grouping Operations, Data Scaling, and Data Binning. Non-traditional machine learning methodologies for the development of the FL subsystem include Shallow Neural Networks (SNN), Deep Neural Networks (DNN), and Convolutional Neural Networks (CNN). Methodologies for the development of the DC subsystem include Majority Voting Method (MVM), Support Vector Machine (SVM), *Softmax* Function (Multiclass classifier), and *Sigmoid* Function (Binary classifier). Finally, applicable programing environments for the development of SOI include Python/Keras, MATLAB, Apache Spark, Java, and C/C++.

### Our Contributions

- In short, our contributions can be summarized as follows: We provide a system machine learning-based detection/classification architecture that can be developed to classify the IoT traffic records into either (binary classifier) or five (multiclassifier) classes. Furthermore, we present detailed preprocessing operations for the collected dataset records prior to their use with deep learning algorithms.
- We provide an illustrated description of our system's architecture modules and the machine learning algorithms demonstrating a comprehensive view of the computation process of our IoT-IDCS-CNN.
- We provide a comprehensive revision of state-of-the-art models to handle the problem of intrusion/cyberattacks detection (IDS) using machine/deep learning techniques.
- We present a systematic model, making use of system engineering (SE) techniques, machine learning technology, and the cybersecurity of the IoT systems field to introduce a new comprehensive SE-based design and architecture for the proposed system of interest (SOI).

## Literature Review

In the last decade, IoT has been engaged in a wide range of everyday life applications. It is growing continuously due to evolution of hardware techniques that improve the design area and bandwidth, such as the field programmable gate arrays (FPGAs) and the cognitive radio-based networks to address the frequency under utilization (Khan et al., 2017). The smart features of IoT have attracted professionals from diverse fields to utilize IoT technology in diverse real-life applications (CISCO Corporation, 2016). However, due to IoT heterogeneity and the restricted resources of IoT devices (limited processing, communication, power, and memory), several IoT security challenges and cyberattacks have emerged with the implementation of such smart technologies and applications. Consequently, the security systems need to be tailored to tackle the security threats of the constrained architecture. Therefore, immense efforts to handle the security issues in the IoT model have been made in the recent years. Many of them were developed by coupling the field conventional machine learning techniques with the cybersecurity field. Indeed, a few promising state-of-the-art experiments were conducted for cybersecurity using deep neural networks models.

The application of conventional and traditional machine learning approaches to solve cybersecurity issues was apparent in many state-of-the-art works. For instances, Hodo et al. used a Multi-Layer Perceptron (MLP) as a member of the Artificial Neural Networks (ANN) family for offline binary classification (normal or attack) (Hodo et al., 2016). The proposed neural network model was trained using traces of IP packets, and subsequently evaluated on its capability to prevent DDoS attacks. The simulation results demonstrated 99.4% accuracy in detecting various DDoS attacks. However, this research focused only on a small dataset that contains one category of attacks (DDoS), which is not realistic as IoT devices/gateways are vulnerable for several groups of attacks, such as probes, R2L, U2R, and others. Similarly, Amouri et al. employed the supervised machine learning techniques to develop an IDS of the IoT communication systems (Amouri et al., 2018). The proposed system tries to learn the normal actions of the nodes and accordingly detect any abnormal actions (anomaly) into the data traffic of the communication network. Their stated simulation outcomes showed their technique's ability in recognizing benign and malicious network traffic. Although the detection of IoT attacks was reasonable, however, the authors have evaluated the system performance over simulated network with a small array of attacks and devices.

Another related research the authors focused on gathering and evaluating the most common cyberattacks was launched over the IEEE 802.11 standard (Kolias et al., 2016). They ended up with a freely accessible dataset encompassing a wealthy mix of benign and malicious records against IEEE 802.11 networks. To do so, they have compiled the Aegean Wi-Fi Intrusion Dataset (AWID) in a real-time utilization communication to maintain a realistic content of the dataset. To confirm the efficiency of their proposed dataset, they applied extensive experimental analysis for different wireless technologies, including Wireless Fidelity (Wi-Fi), Worldwide Interoperability for Microwave Access (Wi-MAX), Universal Mobile Telecommunications System (UMTS), and Long-Term Evolution (LTE) since they confirmed to have comparable cyberattacks by employing various machine learning techniques (Kolias et al., 2013). Their experiments indicated a robust machine learning detection algorithm, especially when implemented using Random Forest and J48 classification algorithms with overall accuracy spans from 89 to 96%. Nevertheless, the manual selection for data features requires long-time processing and can be extremely tiresome.

Jan et al. proposed a lightweight intrusion detection approach, employing an ML-based SVM technique to identify cyberattacks against IoT network using two or three features (Jan et al., 2019). For benchmarking purposes, they compared their proposed intrusion detection classifier model with other ML classifiers, including K-NN and DT methods, to demonstrate the improvement of their model over other model classifiers. Their experimental findings indicated that their proposed IDS model can provide high detection accuracy with satisfactory execution time. In a similar research tendency, Ioannou and Vassiliou have also employed SVM learning to detect abnormalities in IoT networks (Ioannou and Vassiliou, 2019). Accordingly, the developed SVM-based IDS generates its normal profile hyperplane based upon both normal and anomaly activities of the local sensor since it uses the actual IoT traffic. As a result, they reported that the proposed detection model attained around 81% accuracy if implemented with anonymous topology.

Shukla presented a new centralized detection model for IoT intrusions using three different machine learning techniques, including the K-means method (unsupervised), Decision tree (supervised) and a Hybrid model comprising both methods (Shukla, 2017). The experimental results demonstrated that the approaches have attained detection rate ranges of 70–93, 71–80, and 71–75% for the K-mean method, the decision tree (DT) method, and the hybrid method, respectively. However, the hybrid method recorded the highest accuracy as it significantly reduces the false positive factor over the other two detection methods. The system evaluation was performed using small-scale simulated network (16 nodes) with different network topologies.

The application of deep machine learning approaches provided only few promising state-of-the-art research's findings for cybersecurity. One noticeable centralized detection model for IoT intrusions was suggested by Niya et al. in which they applied the deep-learning techniques on the commonly used dataset for IoT attacks, namely, the NSL-KDD dataset (Niyaz et al., 2016). As a preprocessing stage, they encoded the training data of the NSL-KDD dataset using the sparse-auto-encoding mechanism. In this research, the authors developed self-reliant unsupervised deep learning system that has been employed using the encoded training records (i.e., input features). At the classification stage, the authors used a binary classifier using the acquired features along the labeled test dataset that classifies traffic as normal or attack. Finally, to evaluate the efficiency of their implemented system, the authors applied the n-fold cross-validation mechanism and, accordingly, reported their attained findings for accuracy and detection rate measures. However, their obtained results seem rational and comparable.

Brun et al. proposed an IDS for network attacks by employing a machine learning approach using a shallow random NN (Brun et al., 2018). The proposed system relied on packet features that are related to specific cyberattacks, which limits the performance of the system when it is applied with more generic attack features. The developed system has fairly detected cyberattacks of the network traffic with reasonable accuracy. However, the system validation was poorly performed on a validation model comprising of no more than three devices, and simple cyberattacks were engaged. Similarly, Thing studied the cyberattacks and challenges affecting the IEEE 802.11 networks, focusing on the novel attacks in which have never been confronted by the scheme of attack detection (Thing, 2017). Accordingly, the contributors proposed a self-taught deep NN approach to identify and classify network anomalies for the incoming attacks. The proposed approach was built through the Stacked Auto-Encoder (SAE) architecture three hidden layers (256/128/64 neurons, respectively). Their approach considers a multi-classifier with four classes for network traffic, including benign traffic, impersonation attacks, injection attacks, and flooding attacks. Their detection technique attained 98.6% of detection accuracy. However, their proposed scheme lacks to include some other known attacks that severely impact the network performance and security, such as the probes and DDoS. Additionally, the approach still requires a ponderous work and consumes a significant time for feature extraction to reach quite well-classification accuracy levels.

Another appropriate related research can be recalled where the authors constructed their deep learning approach utilizing autoencoders (AE) for anomaly detection (Sakurada and Yairi, 2014). In this work, the normal traffic of the network has been trained using AEs with a dynamic feature engineering mechanism. As a result, the authors concluded that normal traffic data have recorded small reconstruction loss (error) in the dataset of validation, while a larger loss has been recorded for anomalous traffic data of the same dataset. Roopak et al. proposed the deep learning model to detect DDoS attacks over IoT networks (Roopak et al., 2019). They have evaluated their model using the “CICIDS2017” dataset for DDoS attack detection. As a result, they have reported the highest detection accuracy between 86.25 and 97.16%. However, their evaluation stage encompasses a very small representative sample that does not reflect realistic accuracy in the actual IoT environments. Additionally, it is totally unclear how the authors encoded input features for their neural network.

Furthermore, another DL technique has also been applied by contributors of Li et al. (2015) for detecting malicious codes. The authors proposed a hybrid mechanism that employs auto-encoders to extract the features in addition to multilayer Restricted Boltzmann Machines (RBM) to classify the codes. As a result, they concluded that hybrid deep neural networks recorded better accuracy and a processing period over the use of separate deep belief NN or that using shallow NNs in cyberattack detection applications. However, the dataset used in this research needs to be up-to-date to reflect more rationale results and additional practical cyberattacks. Besides, (Abu Al-Haija and Zein-Sabatto, 2020), Al-Haija et al. (2021) presented a novel deep-learning-based detection and classification system for cyberattacks in IoT communication networks employing convolutional neural networks. They evaluated their model, using NSL-KDD dataset scoring accuracy results of 99.3 and 98.2% for the binary-class classifier (two categories) and the multiclass classifier (five categories), respectively. Besides, they validated their system using a 5-fold cross-validation, confusion matrix parameters, precision, recall, F1-score, and false alarm rate. As a result, they showed that their system outperformed many recent machine-learning-based IDCS systems in the same area of study.

Indeed, ML methods have been extensively exploited in cybersecurity of attacks detection and classification in the recent years (Kolias et al., 2013, 2016; Sakurada and Yairi, 2014; Li et al., 2015; CISCO Corporation, 2016; Hodo et al., 2016; Niyaz et al., 2016; Wang et al., 2016; Khan et al., 2017; Shukla, 2017; Thing, 2017; Amouri et al., 2018; Brun et al., 2018; Ioannou and Vassiliou, 2019; Jan et al., 2019; Roopak et al., 2019). A critical restriction is that traditional ML methods need to apply tiresome long-time feature engineering (FE) to attain higher proportions for the detection accuracy of cyberattacks/intrusions. However, the produced schemes are usually not optimum in attaining a high level of validation accuracy for the problems involving the classifiers of multiple classes. Such models do not provide high detectability, especially for the anomaly IoT attacks developed with minor mutations (i.e., slight variations) of previously known attacks. Therefore, the proposed approach of this research is promising to provide better detectability, prediction, and classification for the anomaly attacks developed with minor mutations that are meant to be processed as normal traffic into the IoT environment. It was reported that 99% of the cyberattacks are developed by slightly mutating previously known attacks to generate a new attack, tending to be handled as benign traffic through the IoT network. In fact, even the newly developed cyberattacks depend on the earlier rationalities and notions (Ambedkar and Kishore Babu, 2015). Finally, to sum up, Table 1 compares the research of conventional and traditional machine learning approaches to solve cybersecurity issues.

**Table 1:** A summary of the related research for machine-learning-based IoT security.

**References**	**Method**	**Dataset**	**# of classes**	**Accuracy**
Al-Haija et al. (2021)	Shallow CNN	NSL-KDD	Two-classes	99.3%
Abu Al-Haija and Zein-Sabatto (2020)	Shallow CNN	NSL-KDD	Five-classes	98.2%
Bendiab et al. (2020)	RisNet-50	Zero-day Malware	Two-classes	94.5 %
Shire et al. (2019)	CNN	Zero-Day Malware	Five-classes	91.3%
Baptista et al. (2019)	SOINN	Ransomware file types	Five-classes	94.1
Taher et al. (2019)	ANN + SVM	NSL-KDD	Three-classes	83.7%
Gao et al. (2019)	DNN + Ensemble	NSL-KDD	Five-classes	85.2%
Sapre et al. (2019)	Hybrid	NSL-KDD	Five-classes	78.5%
Jan et al. (2019)	SVM	CICIDS dataset	Two-classes	93.0%
Roopak et al. (2019)	DNN	CICIDS dataset	Two-classes	92.0%
Ioannou and Vassiliou (2019)	SVM	Simulated dataset	Two-classes	81.0%
Brun et al. (2018)	DNN	Real-Time dataset	Two-classes	75.0%
Thing (2017)	DAE	AWID dataset	Five-classes	98.0
Shukla (2017)	NN+KM+DT	Simulated dataset	Two-classes	75.0%
Chowdhury et al. (2017)	CNN+SVM	NSL-KDD	Two-classes	94.6%
Niyaz et al. (2016)	Self-taught learning	NSL-KDD	Five-classes	88.4%
Hodo et al. (2016)	MLP_NN	DoS dataset	Two-classes	99.0%
Kolias et al. (2017)	Hybrid	AWID Dataset	Four-classes	92.0%
Imamverdiyev and Sukhostat (2016)	Extreme LM	NSL-KDD	Five-classes	91.7%
Li et al. (2015)	AE+DBN	KDDCUP dataset	Five-classes	92.0%

## Alternative Architecture Definitions and Architecture Selection

This section involves the study and examination for the various applicable techniques to develop the elements of the proposed system as mentioned earlier; the SOI in this work composed of the following subsystems: Feature Engineering (FE), Feature Learning (FL), and Detection and Classification (DC) subsystems.

### Approaches to Feature Engineering Subsystem

Typically, machine learning techniques entail all input/output records to be numeric. Therefore, all categorical datasets need to be preprocessed and encoded into numerical models prior to the use by the machine learning techniques. The FE subsystem normally composes of several preprocessing and encoding operations to prepare the collected dataset to be safely fed as feature inputs into the next machine learning stage. The proposed FE subsystem consists of three modules as follows:

**Data collection module:** Data collection involves the gathering of information on variables of interest (VOS) within a dataset in a documented organized manner that allows to answer the defined research inquiries, examine the stated hypotheses, and assess the output consequences. In this research, the variables of interest concern with the intrusions/attacks data records over IoT computing environments. Many global datasets of IoT attacks available in the literature can be investigated, including the KDD'99 dataset (Ozgur and Erdem, 2016), the NSL-KDD dataset (Revathi and Malathi, 2013; Canadian Institute for Cybersecurity, 2019), the AWID dataset (Kolias et al., 2016), the CICIDS dataset (CICIDS Dataset, 2017), the DDoS dataset (DDoS Dataset, 2020), the UNSW-NB15 dataset (Moustafa and Slay, 2015), the MQTT dataset (Vaccari et al., 2020), and others.

**Data preprocessing module:** Data preprocessing involves transforming raw data into an understandable and consistent format. Data preprocessing operations are applied as needed at the production stage of the system development life cycle. An example of preprocessing operations includes data grouping/aggregation, such as the grouping by sum, data normalization/standardization, data scaling, and others. Thereafter, proper encoding is typically applied after the preprocessing to provide dataset-features mapping. Only after this stage the data are converted to features output and can be efficiently used by the next feature learning subsystem. Therefore, following, we reviewed the most common preprocessing operations that might be applied (as needed) on the input dataset:

- **Data grouping operations (DGO)** – The main aspect of grouping operations is to determine the aggregation technique for the features/data. Group operations, such as average, max, and sum functions, are usually convenient options (Sarkar, 2018).
- **Data binning operations (DBO)** – The idea of data binning is to band together a set of continuous values into a smaller number of “bins” (Sarkar, 2018). The main goal of data binning is to make the system more powerful and prevent overfitting; however, it has a cost to the performance.
- **Data scaling operations (DSO)** – Usually, numerical datasets do not have a certain numerical range, such as age and income columns (Sarkar, 2018). However, machine learning techniques are based upon distance computations, such as k-Nearest Neighbor (K-NN), or k-Means (K-M) require the input features to be scaled continuously. Typically, three popular methods of scaling are used in machine learning, including normalization, standardization, and log transform. The normalization process scales all values in a fixed range between 0 and 1. The standardization scales the values while considering standard deviation.
- **Data naming/renaming** – This process is usually performed for the data columns of raw dataset records that are required for further processing, utilizing the name of the column such as the encoding techniques that require the name of the column being encoded. For example, sorting data into a table requires the determination of the reference column name for operation.

**Figure 3:**
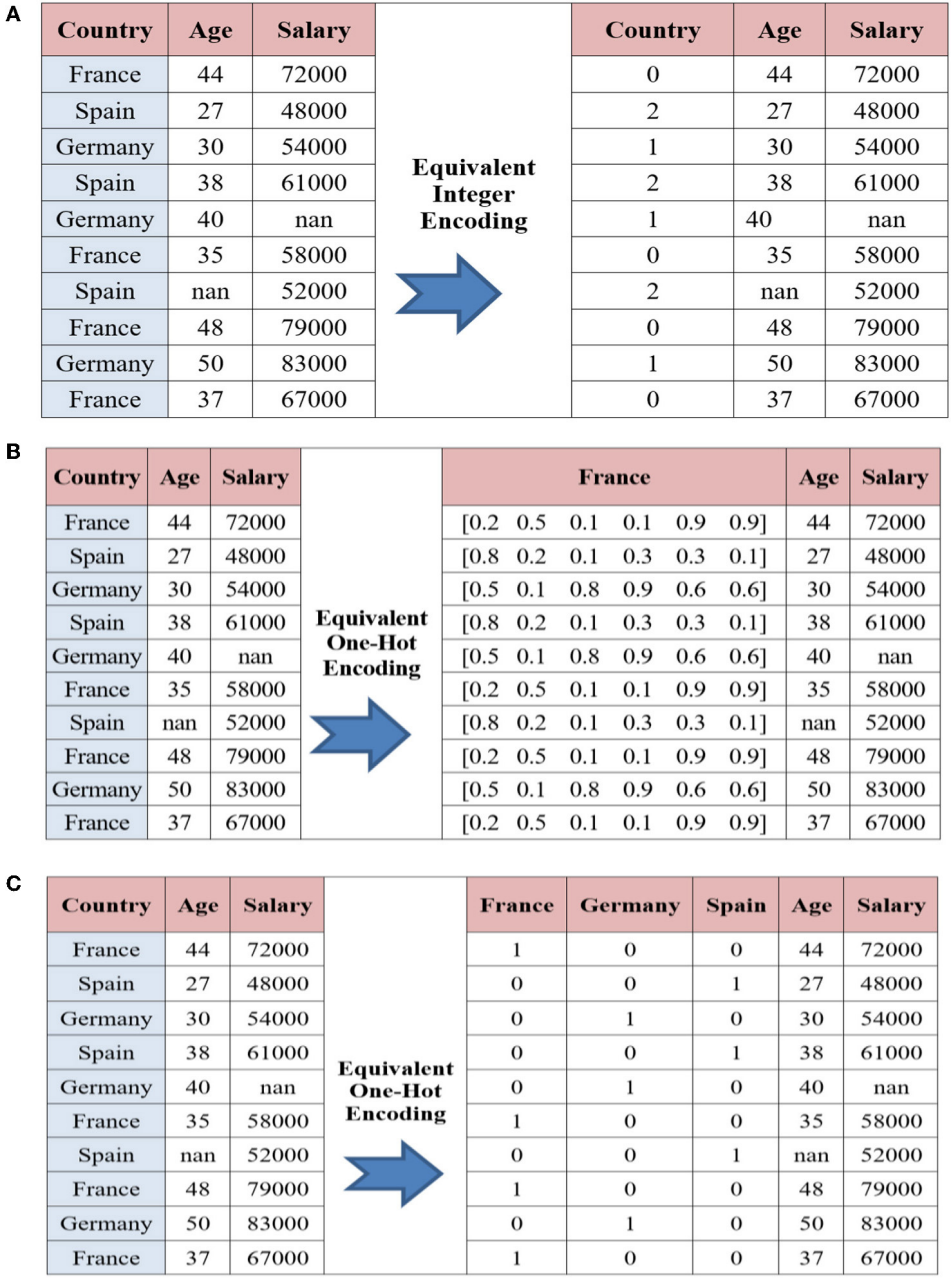
**(A)** An integer encoding example of three categories **(B)** A learned embedding encoding example **(C)** One hot encoding example on City Country.

**Dataset encoding module:** In many real machine learning applications, the dataset will encompass categorical data records. However, most of the machine learning algorithms cannot handle categorical variables unless they are converted to numerical values, and many algorithms' performance varies based on how categorical variables are encoded (Wang, 2019). Categorical variables can be divided in two categories: nominal (has no particular order of data) and ordinal (has some kind of ordered data). Each of which has its own encoding algorithms. In this research, we are employing the NSL-KDD dataset, which is a nominal categorical dataset that needs to be numerically encoded before it can be used in the machine learning module. Three common alternative techniques that are identified for the development of the encoding module for our nominal categorical dataset (i.e., NSL-KDD) are as follows:

- **Integer encoding (INE)** – In this encoding technique, each category is assigned a value from 1 through *K* where *K* is the number of categories for the feature (Brownlee, 2019). INE is where each unique label is mapped to an integer. The problem in this method is that categories that have some ties or are close to each other lose some information after encoding. Also, while the same column contains different numbers for its records, the model would misinterpret the data that will be in some sort of order (0 <1 <2). To overcome such a problem, we used one hot encoder. An example of this encoding method is provided in Figure 3A.
- **Categorical embedding encoding (CEE)** – An embedding-vector is a 1-D array interpretation of categorical data records (Mishra, 2019). For instance, the names of Europe countries can be declared as vectors, each of six floating-point values. LEE is where a distributed representation of the categories is learned. However, the categorical embeddings usually perform pretty good as they have a perception of similarity and difference between themselves, which improve the model's generalization (Mishra, 2019). An example of this encoding method is provided in Figure 3B.
- **One hot encoding (OHE)** – One of the most powerful and common encoding methods in machine learning (Sarkar, 2018). In this method, the categorical data, which are challenging to understand for algorithms, are encoded into numerical format features using 1−*K*− encoding policy. One Hot Encoding is where each label is mapped to a binary vector. Indeed, OHE technique generally performs well for encoding the categorical features into discreet features with no particular order of data (i.e., nominal data records). An example of this encoding method is provided in Figure 3C.

In order to select the most appropriate option of the three alternative approaches to the design and implementation of the dataset encoding module of the FE subsystem, we have used three main criteria that are defined as follows:

- The *feasibility* criterion is an evaluation of the capability to tailor the encoding technique to process the input dataset records and provide the desired feature (such as the percentage of target-dependent elements).
- The *simplicity* criterion is an evaluation of the amount of efforts in investigation and development (such as training time and the number of helps frames) of the method to be used in the selected architecture.
- The *functionality* criterion is an evaluation of the capability to construct the module independently without needing a complete structure of the intended SOI.

Accordingly, One Hot Encoding (OHE) can be elected as the most appropriate alternative (Mishra, 2019) for the development of the dataset encoding module of the FE subsystem. OHE provides unique feature encoding for categorical data where no relationship exists between categories such as the NSL-KDD dataset (Wang, 2019). The proper selection for the encoding technique is significantly important in the feature engineering, which affects the learning process performance and accuracy.

### Approaches for Feature Learning (FL) Subsystem

Feature learning is the process that allows a system to automatically determine the interpretations required to identify the distinctive features from the raw data (Bengio et al., 2013). This allows the system to discover the features and employ them to accomplish a certain mission such as classification or prediction. This requires the input data to be computationally convenient to handle. The FL subsystem is considered as the core part of the proposed SOI since all features are learned at this subsystem. However, three machine learning-based design alternatives can be considered at this stage of the SOI, including Shallow Neural Networks (SNN), Deep Neural Networks (DNN), and Convolutional Neural Networks (CNN).

**Shallow neural networks (SNN):** – SNN (non-deep NN) is a term used to describe neural networks that usually have only one hidden layer (Aggarwal, 2018). In short, data introduced to the network go through a single round (a hidden layer) of pattern recognition.

**Deep neural networks (DNN): –** DNN is an NN composed of a set of hidden layers (also called encoders) between the input layer (visible) and the output layer (visible) of NN (Schmidhuber, 2015). DNN can manipulate the relationship between the input and the output for either linear or a non-linear relationship connection.

**Figure 4:**
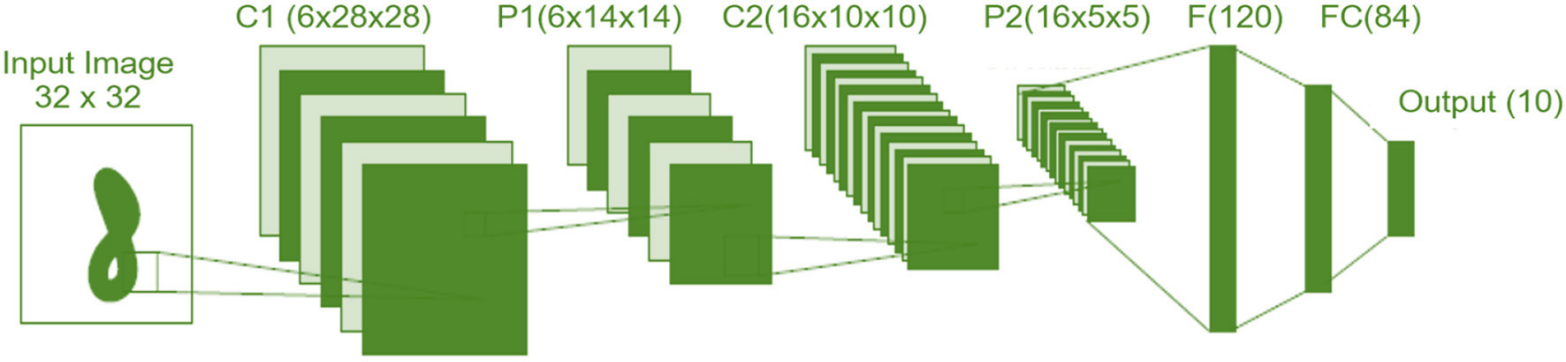
An example of deep convolutional neural network: LeNet-5 (Kim, 2017).

**Convolutional neural networks (CNN):** A CNN is a neural network employing some convolutional layers and some other layers, such as max-pooling and flattening (Li, 2019). The convolution layer applies several convolutional filters to generate a number of feature maps. An illustration example of CNN is given in Figure 4. Specifically, in this figure, we show one of the well-known CNNs that are firstly introduced by LeCun and known as LeNet5 (Kim, 2017). It encompasses of 7 layers as follows: two convolution layers (C1 and C2), two subsampling (pooling) layers (P1 and P2), two fully connected layer (F and FC), and finally end with *softmax* multiclass classification layers with ten categories (classes).

- In order to select the most appropriate option of the three alternative approaches to the development of the Feature Learning (FL) subsystem, we have used four main criteria that are defined as follows:
- The *robustness* criterion is an evaluation of the capability to learn and detect the minor mutations of the input features for the NSL-KDD dataset.
- The *portability* criterion is an evaluation of the capability to train different sets of data with minimum amount of change using learning transfer techniques.
- The *accuracy* is a criterion of an evaluation of the capability of the technique to deliver model-based outputs similar to the real measured output (the percentage of correct predictions/classifications among the total number of predictions/classifications).
- The *functionality* criterion is an evaluation of the capability to construct the module independently without needing a complete structure of the intended SOI.

Accordingly, Convolutional Neural Networks (CNN) can be selected as the most appropriate alternative (Li, 2019) for the development of the feature learning subsystem. CNN provides a comprehensive approach since it requires multiple computational layers each with several parallel feature mapping applications. It can be utilized to learn even a very minor mutation in the input features. However, its performance can be highly enhanced with the utilization of GPU parallel computation.

### Approaches to Detection and Classification Subsystem

**Figure 5:**
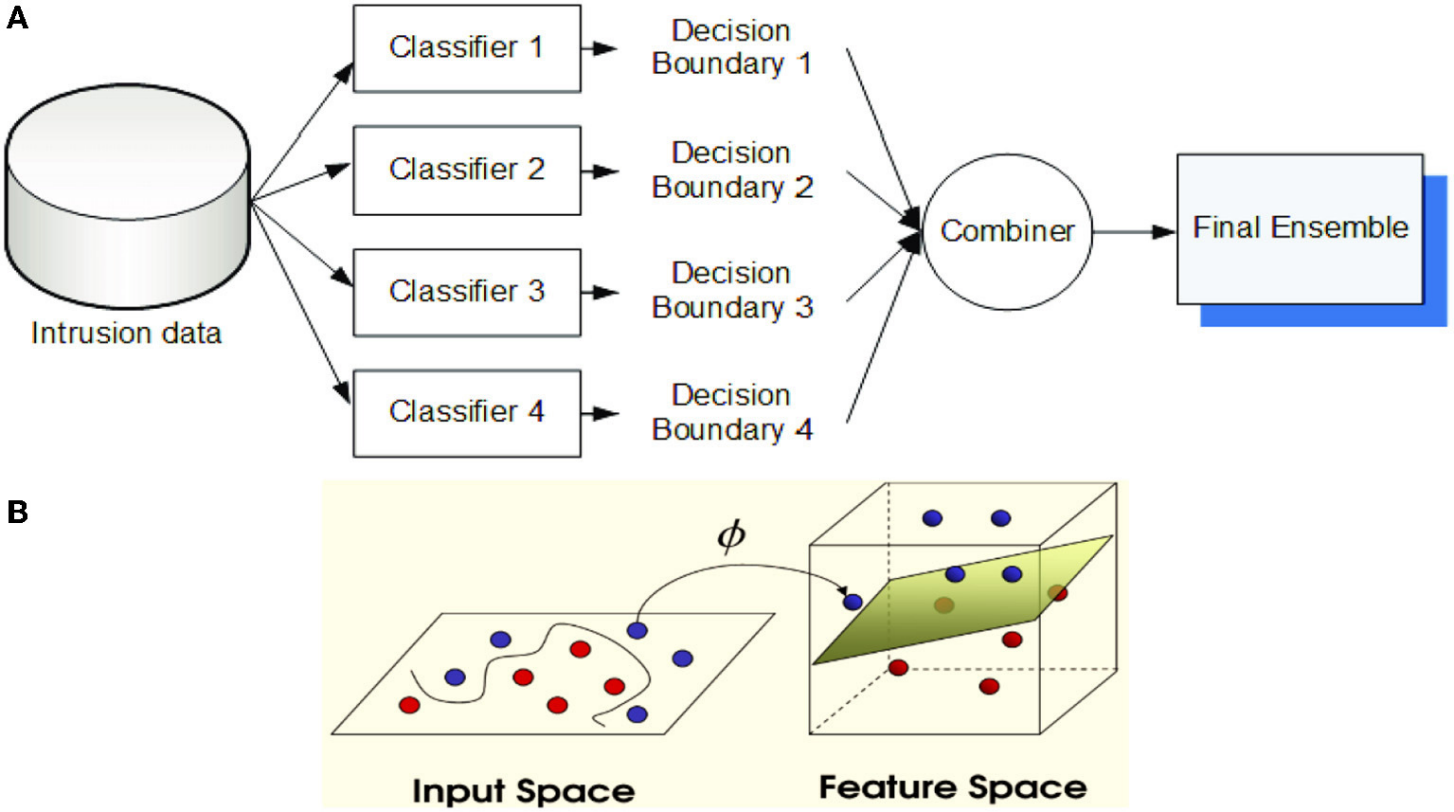
**(A)** Classifier ensemble using majority voting **(B)** The principal idea of support vector machine (Ghose, 2017).

For the DC subsystem, also three alternative design techniques can be considered for this subsystem, including Majority Voting Method (MVM), Support Vector Machine (SVM), and *Softmax* Function Classifier (SFC). Furthermore, the common approaches recognized for the implementation of detection and classification (DC) subsystem that is briefly described include the following:

**Majority voting method (MVM):** After counting the whole elections (predictions) of every executed experiment, the last election result is decided as the experiment that collected the majority of votes (> 50% of all counted votes) (Tama and Rhee, 2017). In the case of none of the experiment exceeds half of the prediction counts, the ensemble method is said to be instable in predicting this data sample (record). An example of classifier ensemble using majority voting is provided in Figure 5A.

**Figure 6:**
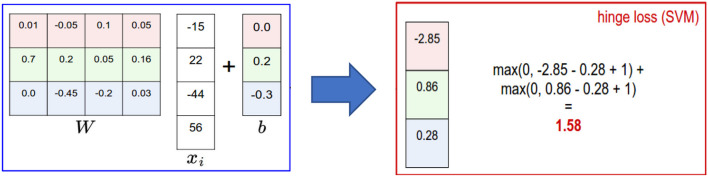
A two-class SVM classifier: An example for one data point using hinge loss (Li, 2019).

**Support vector machine (SVM):** A supervised machine learning approach that is employed for application of prediction and classification (Ghose, 2017). By applying the training records, each sample/record is categorized as belongs to certain category. SVMs are non-parametric technique since they comprise several weighted vectors, nominated from the training dataset, where the number of support vectors is less than or equal to the number of training samples. For example, in ML applications for natural language processing (NLP), it is not unheard of SVMs with tens of thousands of vectors, each comprising hundreds of thousands of data features (Ghose, 2017). Figure 5B illustrates the principle of SVM technique. An example of an SVM classifier is illustrated in Figure 6, which comprises a two-class classification problem (correct class is the blue class). According to the Figure, we compute the same score vector **f**, and then SVM classifier construes these values as scores for the class and its loss function stimulates the correct class to have a greater score over the scores of the other classes. For this example, the ultimate loss value is 1.58.

**Softmax function (multiclass classifier):** is a normalized exponential formula that normalizes a vector of K real numbers (ℝ^*k*^) into a probability distribution comprising of K real number probabilities (ℝ^*k*^) that are proportional to the exponentials of the input numbers (Li, 2019). The *SoftMax* function σ:ℝ^*k*^ ↦ ℝ^*k*^ is defined as follows:


(1) $$\begin{array}{l} {\sigma \left(x\right)}_{i}\;=\;\frac{e^{x_{i}}}{\sum_{j\;=\;1}^{K}e^{x_{i}}}\;for\;i\;=\;1,\;2,\;3,\;\ldots ,\\ \;K\;and\;x\;=\;\left(x_{1},\;x_{1},\;\ldots ,\;x_{K}\right)\in {\mathbb{R}}^{k} \end{array}$$

**Figure 7:**
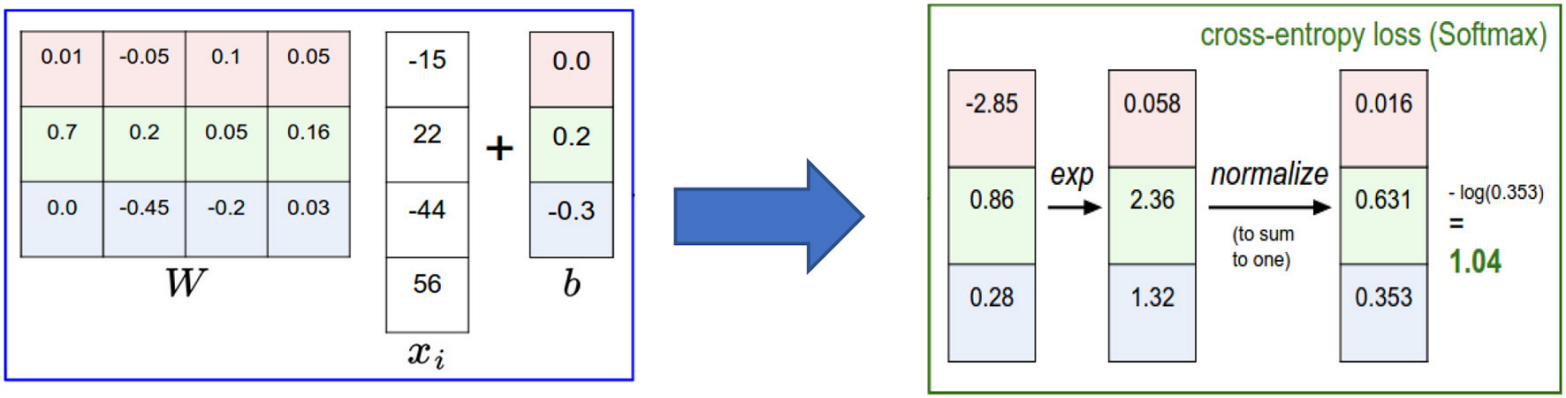
Multiclass SVM classifier: An example for one datapoint using cross entropy loss (Li, 2019).

An example of the *Softmax* classifier is illustrated in Figure 7. According to the figure, we compute the same score vector f, and then the *Softmax* classifier construes these values for each class as unnormalized log probabilities and then stimulates the normalized log probability of the correct class to be higher than the probabilities of the other classes. For this example, the ultimate loss value is 1.04.

As a special case of *Softmax* function, ***Sigmoid*** Function (Binary classifier) – a mathematical function (also called logistic function) that takes a vector of K real numbers as an input and normalizes it into a probability distribution, including of two probabilities (e.g., normal traffic vs. attack) (Li, 2019). The *sigmoid* function *S*:ℝ^*k*^ ↦ ℝ^*k*^ is defined as follows:


(2) $$\begin{array}{l} S{\left(x\right)}_{i}\;=\;\frac{1}{{1\;+\;e}^{-x}}\;=\;\frac{e^{x}}{e^{x}\;+\;1\;}for\;i\;=\;1,\;2\ldots ,\\ K\;and\;x\;=\;\left(x_{1},\;x_{1},\ldots ,\;x_{K}\right)\;\in \;{\mathbb{R}}^{k} \end{array}$$

**Figure 8:**
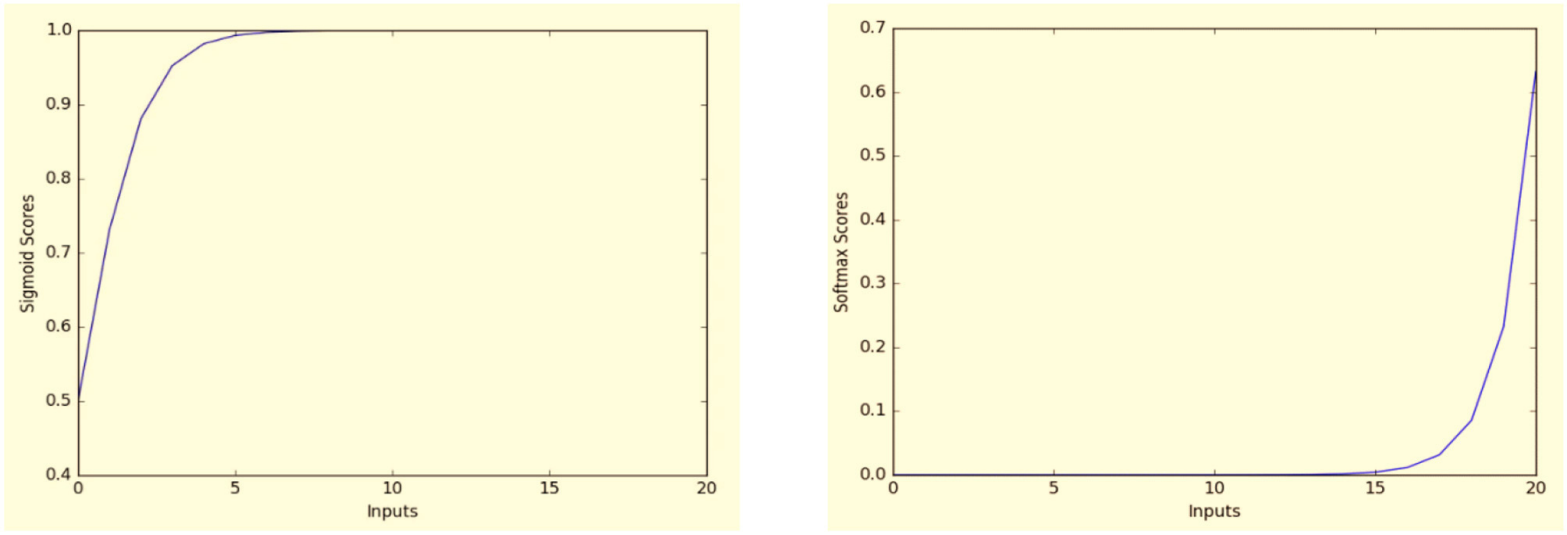
The fundamental property of *sigmoid* and *softmax* functions.

Many other functions are also used to classify the output for neural network such as *rectifiers*, tanh, *Maxout*, and others (Kim, 2017). However, the reason we mentioned only *Sigmoind* and *Softmax*, is that they were both used extensively in several machine learning classification applications. Figure 8 shows examples of applying *Sigmoid* and *Softmax* functions for data inputs *x* = (0, 1, 2, 3, …, 20).

In order to select the most appropriate option of the three alternative techniques for the development of the Detection and Classification (DC) subsystem (i.e., the traffic classification), we have used four main criteria, which are defined as follows:

- The *precision* criterion is an evaluation of the capability to provide precise normalized percentages for the desired outputs.
- The *portability* criterion is an evaluation of the capability to generate the output in multiple classified categories with minimum amount of loss (different numbers of classes).
- The *feasibility* criterion is an evaluation of the capability to tailor the SFC classification technique to accommodate the output parameters for the CNN preceding it.

**Figure 9:**
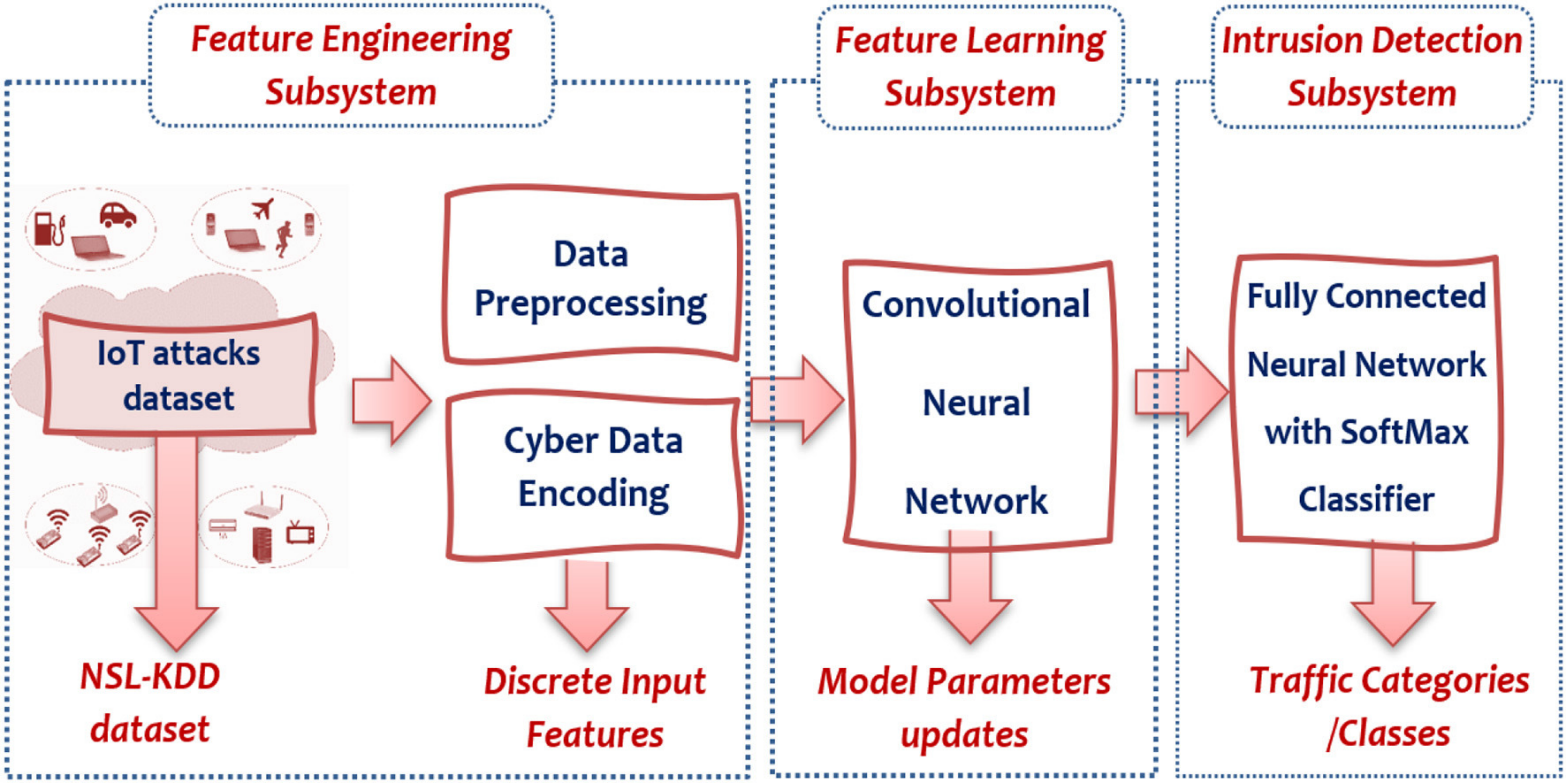
Complete architecture of proposed system of interest (SOI).

Accordingly, *Softmax*Function Classifier (SFC) can be selected as the most appropriate alternative (Tama and Rhee, 2017) for the development of the intrusion detection and classification subsystem. SFC uses the cross-entropy loss, which normalizes the input value into a vector of numbers, following probability distribution that sums up to one (Li, 2019). The output results are in the range [0 ~ 1]. This is highly preferred since we can avoid using binary classification as we are able to accommodate as many classes as needed in our neural network architecture. Finally, the complete architecture of the proposed system comprising all selected subsystems is illustrated in Figure 9.

According to the figure, at the beginning, the raw IoT traffic dataset received from IoT devices is collected to be preprocessed and encoded as input features. This stage is very important (i.e., feature engineering) since it is responsible for utilizing the data knowledge realm to produce distinctive features that permit ML techniques to function appropriately. These produced features present input data to the learning-based convolutional neural network subsystem. This subsystem is responsible for learning and parameters updates (weights and biases) through several computation layers, such as a convolution layer and other hyper-parameter operations. Thereafter, the detection subsystem is formed by two or more layers of fully connected neural network (FCNN) with the last layer containing a few parallel neurons corresponding to the number of classes (categories) for final classification (e.g., attack or normal).

## System Engineering Lifecycle

**Figure 10:**
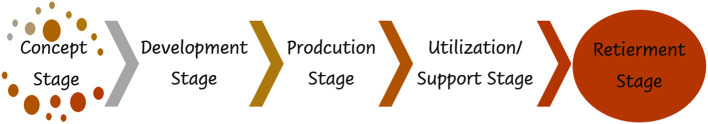
Conventional system engineering life cycle phases using ISO/IEC15288.

Unlike traditional engineering paradigms originated form Archer's “Design Process Model (DPM)” [such as P&C Paradigm (Plug and Chug) and SDBTF Paradigm (Specify-Design-Build-Test-Fix)], which often goes in an endless loop resulting in schedule and cost overrun (Wasson, 2017), System Engineering (SE) entails every system design to adhere to the standardization of system life cycle stages. Therefore, our proposed *ML*−*IoT*−*CovNet* system follows the generic System/Product Life Cycle Model of *ISO*/*IEC* 15288:2008 (ISO/IEC 15288:2008, 2008). In Figure 10, we recall the ISO/IEC15288:2008 model followed in this work.

### System Operation

The system operation phase concerns with operating system to satisfy user needs and provides sustained capability. This stage begins when the system user officially approves the system deployment and ends when the phase-out stage occurs (Wasson, 2017). The proposed system is intended for operation by academic scholars and PhD candidates/researchers involved in the development/upgrading of intelligent solutions/systems for IoT security against the different types of cyberattacks for academia and industry. Such development can be targeted and supported mainly by the governmental and industrial organizations for information technology and security. The proposed system once deployed - to begin active duty - in its current status, it could be run for 3 consecutive years with minor and regular adjustments in the configurations of the system's environment. During the operational lifetime of the system, refinements and improvement updates may be obtained and configured to enhance the capabilities and functionalities of the target system in support of its planned missions in detecting and classifying the data traffic received by IoT networking systems. One major example of system upgrades is to have a timely-based training process for the *CovNet* network of our SOI with newly developed IoT-attacks and threats (for example, if the rules of an existing IDS will be updated). Indeed, these incremental upgrades keep the system in its full operational capability (FOC) (Wasson, 2017).

### System Maintenance

**Figure 11:**
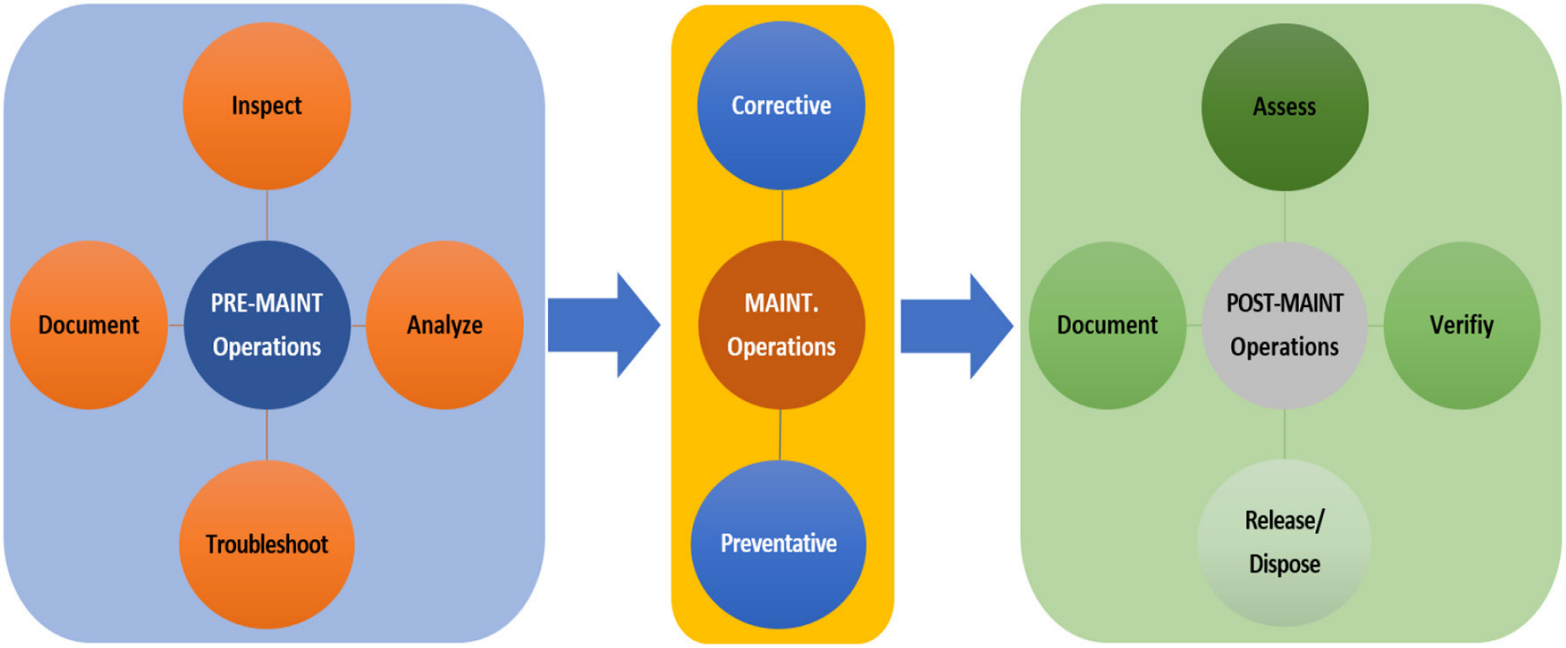
A system maintenance plan for our SOI.

The system maintenance phase concerns ensuring the optimal efficiency and availability of the proposed SOI, and it comprises a set of actions and tasks required to correct the deficiencies/defects that may impact mission success of the SOI. System maintenance is ultimately important as it maximizes the system reliability, availability, and performance and minimizes the failure cost (money/time) as well as reduces the risk of components breaking down. Once the system maintenance is effectively preformed, the system is resumed to its active service/operation phase. To ensure improved maintenance operation, the system maintenance plan usually includes pre-maintenance operations, maintenance operations, and maintenance operations as illustrated in Figure 11.

**Figure 12:**
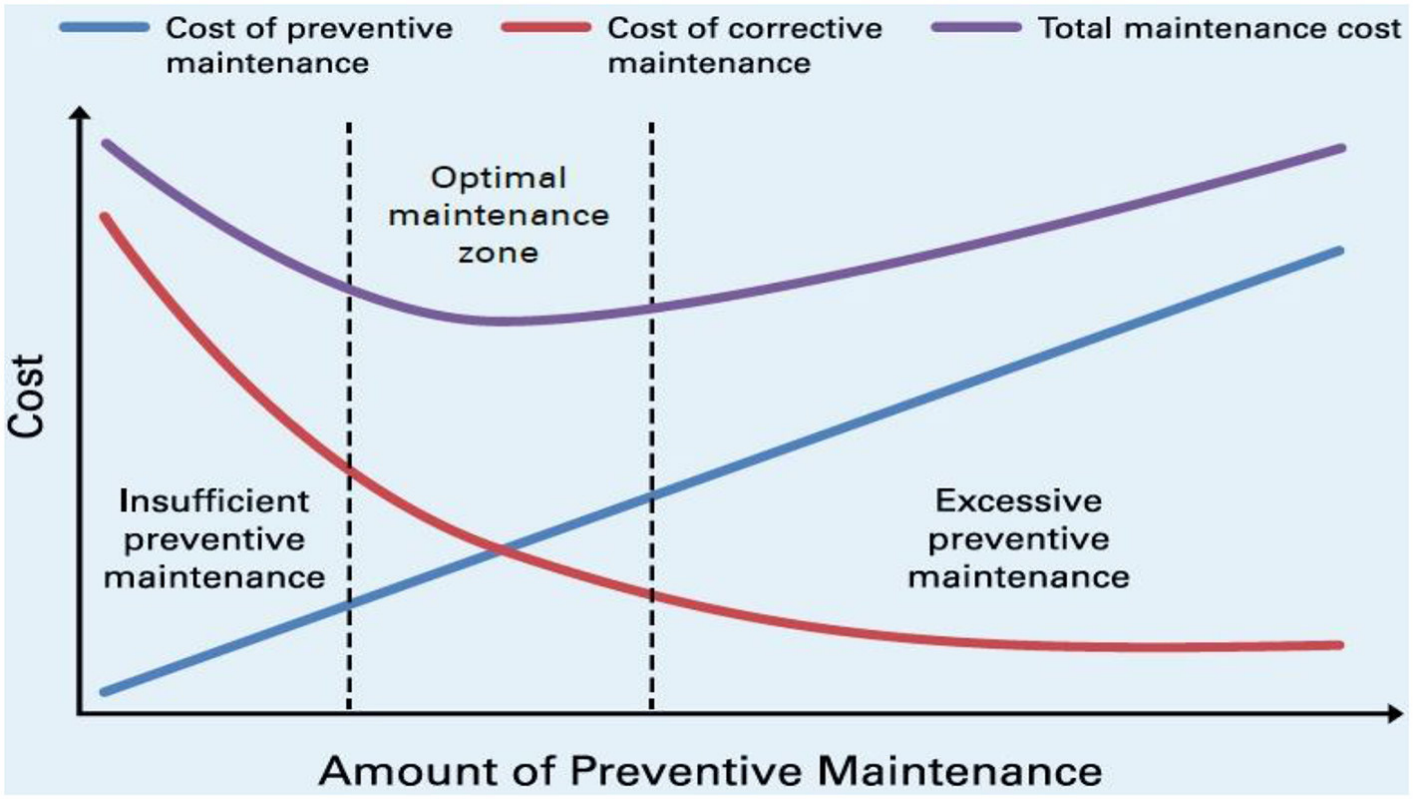
Total maintenance cost and optimal maintenance zone (Bachir et al., 2017).

Finally, to ensure improved optimization and higher reliability of SOI, it is important to find the optimal sense of balance among preventive maintenance (PM) and corrective maintenance (CM) while satisfying the goal and objectives of the SOI mission. Figure 12 illustrates the impacts of frequent maintenance on the costs, PM, or CM (Bachir et al., 2017). The optimal amount of preventive maintenance occurs when the costs of corrective and preventive maintenance meet.

For the proposed SOI, the system maintenance operations can be performed at a user/operator level and at the organizational level as well. This can be performed through preventive maintenance and corrective maintenance actions.

**Preventive maintenance**: regularly performed on the working component of the system (equipment/software) to minimize the probability of a component's failure (by enhancing the component's Mean Time To Failure - MTTF). PM activities are performed to retain the SOI at a prescribed degree of performance (Blanchard and Fabrycky, 2006). Indeed, the preventative maintenance is ultimately important as it decreases downtime of the system. Also, such maintenance can be performed as time-based preventive maintenance or usage-based preventive maintenance.

**Figure 13:**
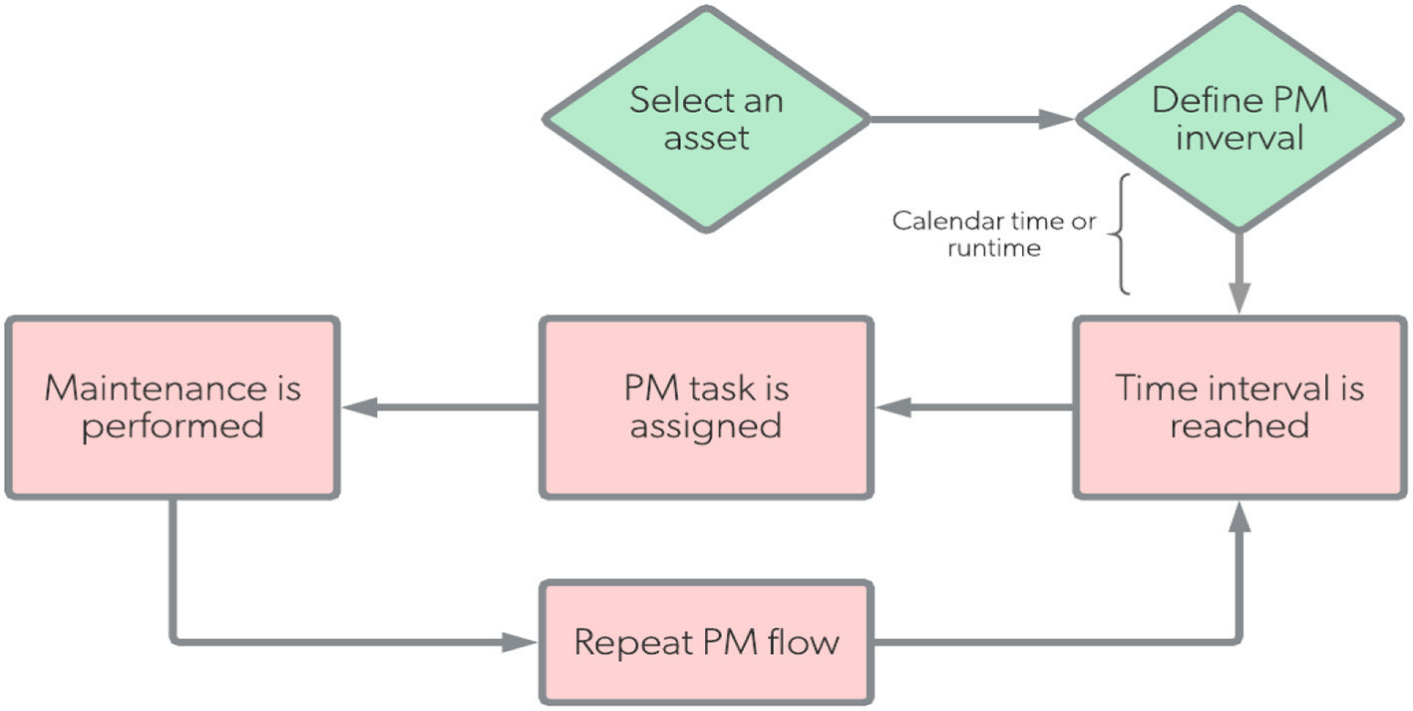
Preventive maintenance workflow for our SOI.

In our developed SOI, we planned our preventive maintenance (PM) using time-based preventive maintenance to be performed according to the workflow diagram given in Figure 13. For our system, the privative maintenance would be performed in case of any problem occurred in SOI or its platform environment, such as:

— It is essential to have PM that provide both logical security (such as installing a licensed powerful antivirus system) and physical security (such as having the system in a safe process room that can only be accessed by authorized users and tighten all cable connections).
— It is planned to keep a regular inspection on the Nvidia GPU component as it severely impacts the system performance and availability in the event of a breakdown. Indeed, all system hardware should be inspected regularly.
— As preventative maintenance, it is very recommended to maintain a mechanism of standby redundancy for the sensitive components in the system, such as the GPU and CPU, as well as the SOI's software system to be replicated on an external storage drive.
— Also, since our system is a software system developed by the MATLAB platform, time-based preventive maintenance is required to check for updated libraries and built-in functions of MATLAB system as well as the corresponding toolboxes, such as the deep learning and parallel computing libraries.
— Moreover, it is part of the preventative maintenance plan to track the annual license for the MATLAB and other software packages utilized in this SOI.
— Furthermore, it is crucial to develop a regular backup plan for the system configuration and data utilizing off-site storage and cloud-based strategies.
— In addition, to avoid the common power problems, such as blackout or voltage surge, we recommend having a standby Uninterruptible Power Supply (UPS) to resort to battery backup power.

**Corrective maintenance:** on-demand performed on the faulty component of the system (equipment/software) to restore broken down systems. This type (also called breakdown maintenance) of maintenance is performed to restore the SOI to its originally prescribed operational state within minimum amount of maintenance time (the component's Mean Time To Repair - MTTR) (Blanchard and Fabrycky, 2006). Upon detecting a faulty component, corrective maintenance should be planned and scheduled for a future time; otherwise, the problem might become an emergency maintenance need (leads to interruption in service). During the execution of corrective maintenance work, the component is repaired, restored, or replaced. Indeed, the corrective maintenance is also ultimately important as it decreases downtime of the system.

**Figure 14:**
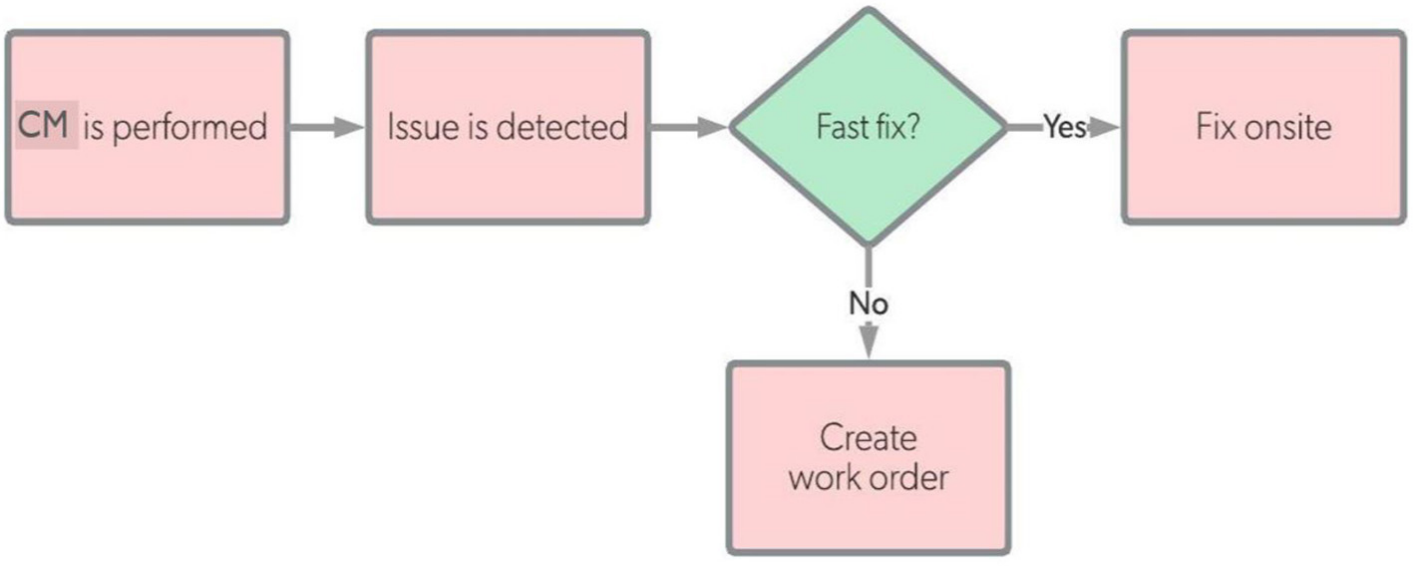
Corrective maintenance workflow for our SOI.

In our developed SOI, we planned our corrective maintenance (PM) upon demanded to be performed according to the workflow diagram given in Figure 14. Once an issue/problem occurred, the appropriate corrective maintenance should be performed as soon as possible to fix the issue either on-site (for minor issues) or off-site (for major issues). For our system, the corrective maintenance would be performed in case of any problem occurred in SOI or its platform environment, such as:

— Diagnose and fix the logic errors in the SOI functionality, appearing during the system operation such as misdetection of present data.
— Restore the proper backup configuration settings, in case of change system environment change or corrupt such as the degrade in response time.
— Debugging the SOI program coding, troubleshooting, and fixing to avoid syntax and semantics errors. This also includes the update of system drivers, especially the drivers for GPU cards.
— Replace defective hardware components, such as Memory, GPUs, networking connections, and others, with minimum possible MTTR.
— Fixing any power failure problems, especially during the period using the UPS in order to enable the main power supply.
— Upgrade or replace any outdated hardware or software components to avoid the deficiency effect on other parts of the system.
— Perform maintenance on software service modules that are running slowly to bring it back to its optimal performance.

### System Disposal

At a certain moment, any deployed system will turn out to be no longer useful, wasteful to maintain, outdated, or unrepairable. Therefore, the system disposal is an essential phase of the system life cycle. The system retirement (disposal) stage involves a set of actions and tasks needed to terminate the current system from its active operation. It begins when the stage of phase-out appears and preceded by the deactivation stage of the system. Throughout the system retirement phase, the existing system can be retired and dispositioned from its active operation *via* different techniques, including selling, leasing, storage, or disposal. Also, the system disposal phase can be altered by several replacements policy, including disassembly, destruction, burning, and burial (Wasson, 2017). Finally, the disposal stage of the system might also involve ecological restoration and renovation to reconstruct the system's field site or retirement region to its normal status (Wasson, 2017).

In our developed SOI, the principal plan of the disposal phase is completely uninstalling the software system from the site with all associated toolboxes, packages, and data. Besides, the disposal phase should also consider:

— Strategies to discard system information hardware and software. This includes details of underlying hardware (such as the GPU/CPU type, specifications, and others) and the system drivers as well as the user and system data.
— Plans to move to a new system. The required information can be transferred, abandoned, ruined, or filed. Also, further considerations are needed for restoring the archived information in the future such as the utilization of external archiving/storage platforms involving the use of cloud-based services and standby storage drives.
— Security considerations: It should be stated that the complete implementation of the system disposal stage is vital, since severe faults at this moment might place the organizations/stakeholders at a high chance of revealing confidential data such as the development coding and architecture.

### System Life Cycle Cost

**Figure 15:**
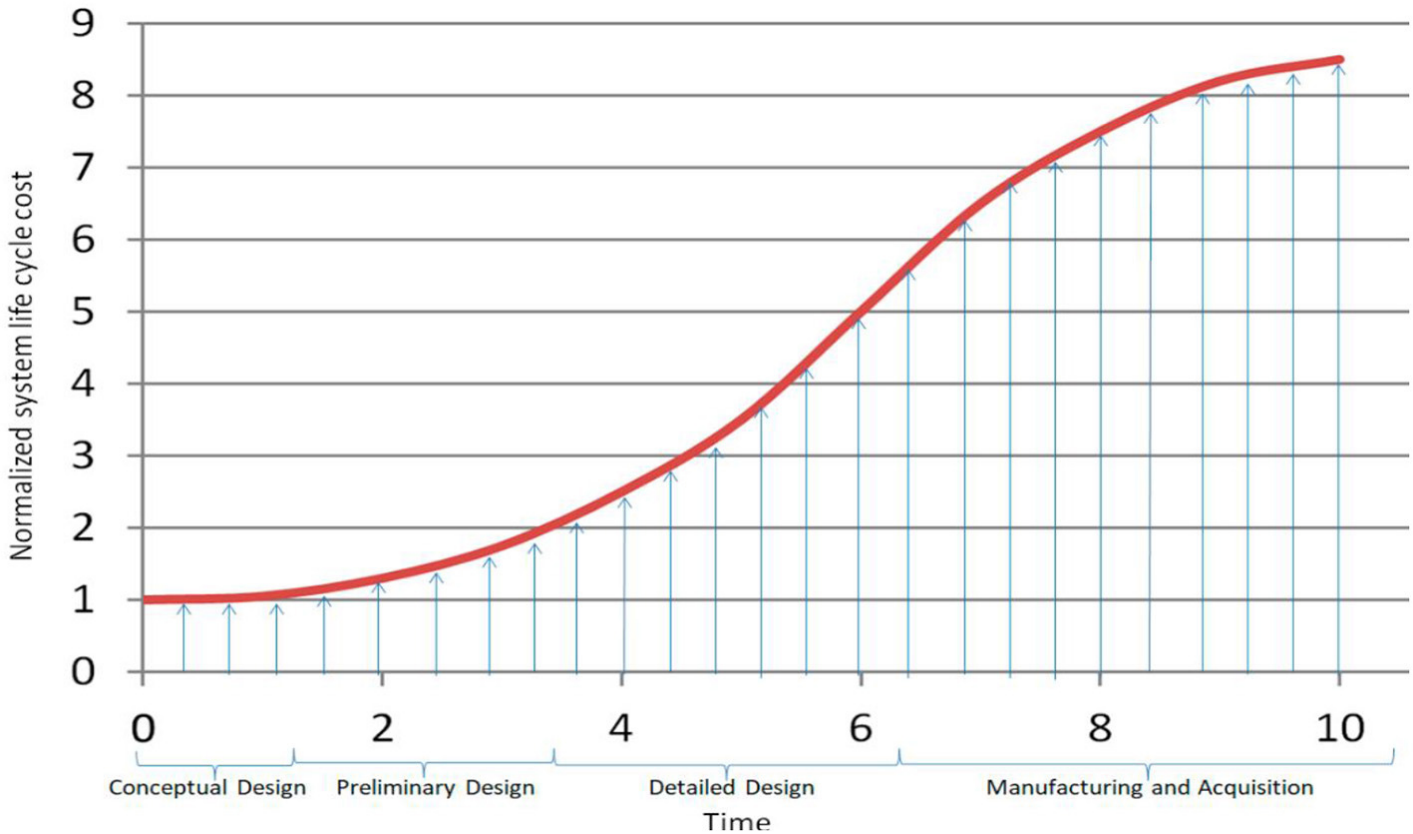
Common SE curve system life cycle cost (LLC vs. Time).

In system engineering (SE), life cycle costing (LCC) concerns with investigating and assessing the system cost. LCC is a crucial component for the decision maker of SOI since several decisions are planned based on LCC (Pohl and Nachtmann, 2010). Therefore, it is important that the cost estimations are constructed with maximum accuracy and precision. Like any SE process, estimating LLC is an iterative process where the system's cost estimation can undergo updates as the system progresses throughout the different stages of the system life cycle (SLC). However, SE aims at trade-offs to satisfy requirements, reduce the system life cycle costs, and lessen the risk to a satisfactory point (Wasson, 2017). Figure 15 displays the common cost curve in the system life cycle for conventional SE (Madni and Purohit, 2019). In this figure, the vertical axis shows the system LCC (normalized into 0 ~ 9), while the horizontal axis shows the time factor. According to the figure, the system engineering seems to have lower investment at the initial stages of the SLC (e.g., concept/preliminary stages) while the investment significantly increases at the late stages such as production and operation stages.

**Figure 16:**
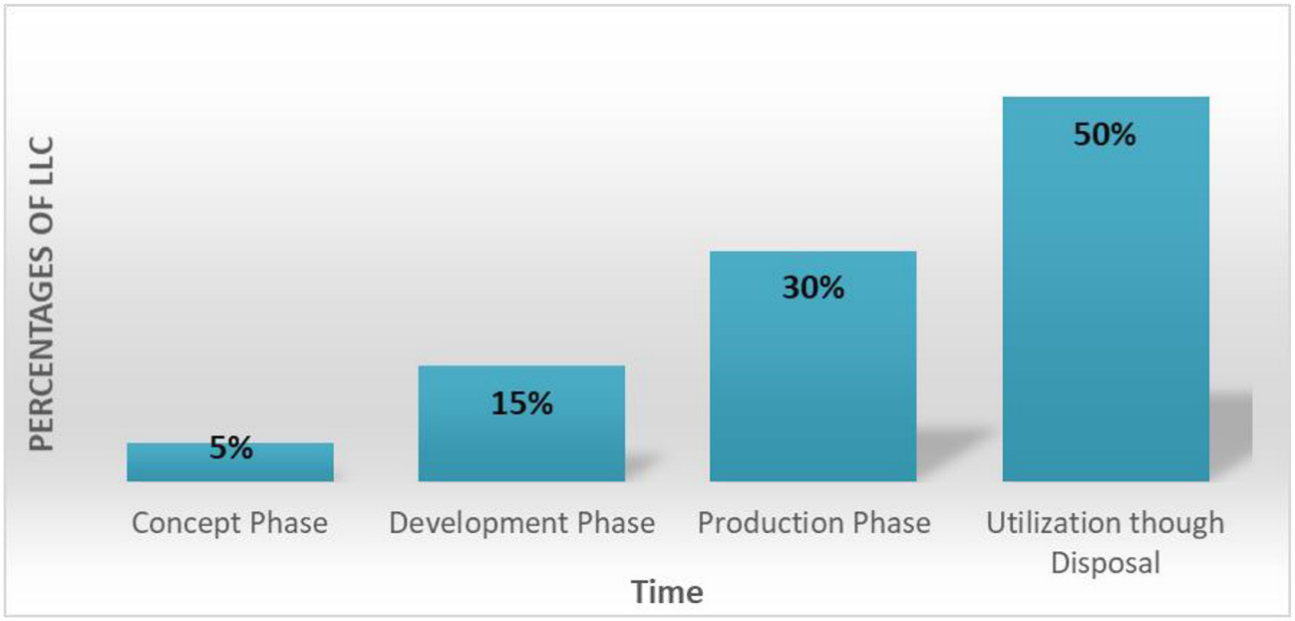
Percentages of life cycle cost with time for proposed system development.

In our developed SOI, we have developed our LLC plan in compliance with SE cost policy, where we lowered the expenditure at the early phases of system life cycle and focused the investment at the later phases. Figure 16 illustrates our LLC policy for our system life cycles. As shown in the figure, concept and development phases collectively account for 20% of the total LCC, whereas the remaining 80% of the LCC was invested in the system production through system disposal phases (this includes testing, operations, support, maintenance…).

## Conclusions

A new efficient and generic top-down architecture for intrusion detection and classification in IoT networks using non-traditional machine learning is proposed in this article. The proposed architecture can be customized and used for intrusion detection/classification incorporating any IoT cyberattack datasets, such as the KDD'99 dataset, the NSL-KDD dataset, the AWID dataset, the CICIDS dataset, the DDoS dataset, the UNSW-NB15 dataset, the MQTT dataset, and others. Specifically, the proposed system is composed of three subsystems: Feature Engineering (FE) subsystem, Feature Learning (FL) subsystem, and Detection and Classification (DC) subsystem. All subsystems have been thoroughly described and analyzed in this article. Accordingly, the employment of deep learning models will enable to detect the slightly mutated attacks of IoT networking with high detection/classification accuracy for the IoT traffic represented by either real-time system or a pre-collected dataset. Also, this work has employed the system engineering (SE) techniques, the machine learning technology, and the cybersecurity of the IoT systems field to introduce a new comprehensive SE-based design and architecture for the proposed system of interest (SOI). The collective corporation of the three fields has successfully yielded a systematic engineered system that can be implemented with high performance and accuracy. Besides, in this work, we have applied SE's top-down approach, where the overview of the SOI is initially formulated and specified through first-level subsystems. Then, each subsystem is refined comprehensively, providing all details of its elements (modules) and their design alternatives. This top-down decomposition simplifies the implementation of each level of the system abstraction with iterative and incremental development, integration, and validation processes. This also provides greater details of each subsystem/module that helps the decision-making authority/stakeholders for system configurations and future change management plans. Moreover, this work emphasizes the benefits of employing the non-traditional machine learning techniques involving CNN networks in the cybersecurity of IoT environment to detect and classify the data traffic of IoT communication. Unlike traditional machine learning techniques used for detection tasks, which require the data to be linearized in 1-D arrays (or vectors) and a feature extractor to be manually designed, the utilization of CNNs for the detection task of IoT cyberattacks requires the dataset to be converted into 2-D matrices to accommodate input for the deep 2D convolution operations. This process helped generate the feature maps that extract unique features of each dataset sample. Note that the nature and the number of extracted features depend on the values and the numbers of convolution filters (kernels) applied over the input data. The filters' values are also called the weights of the convolution layers, and they are defined using the training procedure. Finally, due to the large number of weights (because of many hidden layers) needs to be trained in the deep neural networks, the neural network requires more computations and thus requires longer time for the training process. This disadvantage (i.e., computational load) can be mitigated through the use of high-performance computing platforms involving the use of graphical processing units (GPUs) and efficient programming modules (e.g., mini-batch normalization).

## Recommendation for Future Work

Several recommendations for future research works may be considered to extend this study. These further recommendations can be listed as the following:

— The data records can be the coordinate of the next research study by setting up a real-time IoT communication network with sufficient number of nodes and gateways, incorporating nodes diversity. A further researcher can develop a new software system that catches and investigates any data packet communicated through the IoT environment (in-going and out-going) and come up with attacks to update an existing dataset or to come up with a new dataset. Note that the packet collection and investigation should be performed for a sufficient amount of time to provide more insights into the type of packets (normal or anomaly) processed at IoT networking.
— The proposed SOI can also be tuned and used to perform many other real-life applications, requiring image recognition and classification, such as medical, biomedical, handwritten recognition applications, and others.
— Finally, the proposed system can be employed by an IoT gateway device to provide intrusion detection services for a network of IoT devices such as a network of ARM Cortex-based nodes. More investigation on the proposed SOI can be reported, including power consumption, memory utilization, communication, and computation complexity over the low-power IoT nodes with tiny system components (such as the battery-operated/energy aware devices).

## Data Availability Statement

The original contributions presented in the study are included in the article/supplementary material, further inquiries can be directed to the corresponding author/s.

## Author Contributions

The complete manuscript is the sole work of QA.

## Conflict of Interest

The author declares that the research was conducted in the absence of any commercial or financial relationships that could be construed as a potential conflict of interest.

## Publisher's Note

All claims expressed in this article are solely those of the authors and do not necessarily represent those of their affiliated organizations, or those of the publisher, the editors and the reviewers. Any product that may be evaluated in this article, or claim that may be made by its manufacturer, is not guaranteed or endorsed by the publisher.
